# Symbolic-Based Recognition of Contact States for Learning Assembly Skills

**DOI:** 10.3389/frobt.2019.00099

**Published:** 2019-10-17

**Authors:** Ali Al-Yacoub, Yuchen Zhao, Niels Lohse, Mey Goh, Peter Kinnell, Pedro Ferreira, Ella-Mae Hubbard

**Affiliations:** ^1^Intelligent Automation Centre, Loughborough University, Loughborough, United Kingdom; ^2^Beijing Ewaybot Technology LLC, Beijing, China

**Keywords:** symbolic representation, imitation learning, feature transformation, Piecewise Aggregate Approximation (PAA), K-means, Hidden Markov Model (HMM)

## Abstract

Imitation learning is gaining more attention because it enables robots to learn skills from human demonstrations. One of the major industrial activities that can benefit from imitation learning is the learning of new assembly processes. An essential characteristic of an assembly skill is its different contact states (CS). They determine how to adjust movements in order to perform the assembly task successfully. Humans can recognize CSs through haptic feedback. They execute complex assembly tasks accordingly. Hence, CSs are generally recognized using force and torque information. This process is not straightforward due to the variations in assembly tasks, signal noise and ambiguity in interpreting force/torque (F/T) information. In this research, an investigation has been conducted to recognize the CSs during an assembly process with a geometrical variation on the mating parts. The F/T data collected from several human trials were pre-processed, segmented and represented as symbols. Those symbols were used to train a probabilistic model. Then, the trained model was validated using unseen datasets. The primary goal of the proposed approach aims to improve recognition accuracy and reduce the computational effort by employing symbolic and probabilistic approaches. The model successfully recognized CS based only on force information. This shows that such models can assist in imitation learning.

## 1. Introduction

Industrial robots can efficiently manipulate and assemble objects in a controlled environment with minimum variations. However, they have limitations in assembling parts with geometrical variations and tighter tolerances. In such applications, force signals play a crucial role especially when the robots have to interact with the surrounding environment. Nevertheless, the force signals are noisy and ambiguous to interpret and use (Wen et al., 2014). Humans, on the other hand, can robustly perform assembly tasks with tight tolerances (Park et al., 2008) because they are very efficient at using haptic (F/T) information, especially when vision cannot provide the required information. Consequently, robots can benefit from understanding how humans use such haptic feedback information during an assembly process. This can empower robots to use force and torque with human-like capabilities allowing them to learn and adapt according to the variations in the environment and adjust movement for tight tolerance assembly.

Most of the research work reported in the area of imitation learning is based on visual perception. This is mainly because humans mostly rely on vision to gain adequate information about objects' relative positions and their geometrical properties (Ernst and Banks, 2002; Rozo et al., 2013). In assembly applications, perception importance can vary with motion, where gross motion relies on vision while fine motion requires haptic information, especially in contact situations. The focus of the work reported in this paper is on the use of haptic information to learn an assembly task. Capturing human skills is particularly complicated for assembly processes which often involve an understanding of hidden process features and tacit knowledge. For example, for a successful assembly task, an understanding of various types of contacts between objects and their corresponding forces is required. Another important aspect of an assembly process is the sequential relations between different CSs during the assembly. Henceforth, different skilled operators can perform the stages of the same task with different temporal properties (transition between states and durations). In order to capture, understand and interpret human skills from a number of trials, those trials must be aligned (in terms of duration). Also, the underlying pattern of the haptic information must be extracted to reveal the sequential (temporal) knowledge (i.e., human skill). Hence, those skills must be modeled so that they can adapt to task variations for robotic assembly.

A great deal of research has been conducted on the recognition of the CS. The approaches for CS recognition can be arranged into two groups, i.e., analytical approaches and learning-based approaches. Essentially, the analytical model of the mating system has no single structure. The general model is composed of a set of analytical equations (sub-models), where each equation describes a particular contact state based on a physical analysis of the state. Furthermore, these sub-models usually rely on a set of approximation and assumptions to simplify the given problem. Hence, current analytical approaches to recognize CS is limited in terms of robustness and speed (Jakovljevic et al., 2012). The main limitation of analytical approaches is latency since it relies on a very complex computation (Nuttin, 1995). Learning approaches, on the other hand, appear to be a better alternative when taking the recognition of the CSs into consideration.

Various learning-based approaches to recognize CSs have been presented in the literature. For example, the Hidden Markov Model (HMM) has been implemented to recognize CS based on F/T information in tele-manipulation and result were presented in Hannaford and Lee (1990). However, the proposed models rely on extensive training and are only applicable to large clearance between the assembled parts. In Dong and Naghdy (2007) an HMM was used to recognize the CS of a Peg-in-Hole (PiH) assembly in a virtual environment, and to recognize the CS during the on-line PiH process. However, the accuracy of the trained HMM depended on the accuracy of the virtual world model which generally has nominal behavior. Lau (2003) proposed a framework of CS recognition in industrial robot assembly platform using HMM and F/T information, where it was experimentally proven that HMM-based with F/T is superior compared to the conventional CS recognition (CAD-based and kinematic-based).

Jasim et al. (2017) have developed a method that combines the Expectation Maximization and Gaussian Mixture Model (EM-GMM) to recognize the CS of PiH insertion during an automated process. In Jasim et al. (2017) the number of Gaussian were determined using Distribution Similarity Measure-based (DSM). In this research, the trained GMM models were evaluated using a rubber PiH insertions with two different parts elasticity. Yet, the work reported in Jasim et al. (2017) did not employ feature selection or transformation algorithms in order to reduce the computational effort. A Piecewise Affine Autoregressive Exogenous (PWARX) method has been presented in Okuda et al. (2008) to recognize the CS during the PiH assembly process. The core idea of the PWARX was used to control a robot during the PiH process based on a set of mathematical models (PWARX sets). In this case, the control was achieved by switching between the PWARX models using a Support Vector Machine (SVM). The SVM functionality was to recognize CS and accordingly switch over controllers to select the suitable models for the given CS. The computational power required for this method is quite high (Mikami et al., 2010), and the PWARX model is a complicated model (Nakabayashi et al., 2013). In Jakovljevic et al. (2012) a SVM has been employed to classify two successive states based on pre-designed features sequentially. The selected features were designed based on the quasi-static insertion force model (Whitney, 2004). This method relies on pre-defined features and a complex hierarchical classification algorithm since SVM is only a binary classification approach. This work also relied on designed features which were pre-selected by designers thus making the method less autonomous. Hertkorn et al. (2012) generated a wrench matrix based on the CAD models of the assembly parts with a particle filter to recognize the CS based on the F/T measurements. This method was implemented to resolve the ambiguity of the force measurements and recognize the contact formation of a rectangular workpiece on a flat surface. The drawback of this work was the simplicity of the part's geometries used to validate the proposed approach.

Jamali et al. (2014) presented a CS learning algorithm based on a symbolic representation of temporal behavior during robot valve opening process where force signals were clustered using the Minimum Message Length (MML) (Wallace and Dowe, 2000). The labeled symbolic data were used to train an HMM to recognize the CS. The overall accuracy achieved by this method was 81% about x-axis and 85% for rotation about the y-axis. Nevertheless, the convergence time of the GMM/MML might delay the recognition of the CS. Also, it relies on exploration movements in order to recognize the CS.

Most of the aforementioned research follow pattern recognition in the extracted/selected features by temporal knowledge modeling (capturing). This can be captured in the symbolic or non-symbolic domain. The main advantages of the non-symbolic models are their parametric nature and their capability to capture variations in human skills (Nejati and Könik, 2009). On the other hand, the symbolic approaches are well-known for capturing complex human behavior with simpler and shortened models that have better computational performance. For instance, symbolic approaches can capture the assembly sequence at different hierarchical levels (granularity), which is difficult using probabilistic approaches. Even though symbolic models have traditionally been considered unsuitable for controlling real-world systems (Calinon and Billard, 2008), researchers are now making effective use of these models for skills representation, evaluation, generalization and robot control (Mohammad and Nishida, 2014). These models are computationally efficient, simple, and capable of capturing complex human skills. Therefore, the research work reported in this paper explores the use of symbolic models to capture human assembly skills.

Despite significant progress in the field, researchers have been relying on algorithms which have significant latency. Furthermore, symbolic-based recognition of CS for imitation learning of PiH problems has not been sufficiently explored in the presenters of geometrical variation, in analogy to the material property (elasticity) variation presented in Jasim et al. (2017). In fact, probabilistic models trained based on symbolic representation converges faster than probabilistic models trained based on numeric representation (Kwiatkowska et al., 2004). Thus, it is believed that combining symbolic representation based on a simple segmentation approach [i.e., Piecewise Aggregate Approximation (PAA) or K-means] will result in more computationally efficient CS recognition with comparable robustness and accuracy.

This paper investigates a symbolic-based CS recognition approach which combines feature transformation methods, i.e., Principal Component Analysis (PCA), time-series segmentation, symbolic assignment, data labeling and HMM training, in order to reduce the computational effort required for CS recognition. As a validation example, the PiH assembly was adopted to demonstrate the efficiency of the proposed approach. Despite the apparent simplicity of the PiH assembly, it belongs to the group of parts mating problems that are highly non-linear and difficult process (Chen, 2011; Kronander et al., 2014). The main contribution of this paper is to develop a method that can identify contact states in an actual assembly process, i.e., PiH assembly. The development of this method involves the identification of CS during the PiH process based on symbolic representations of the force/torque signals (non-vision information). In addition to that, the relation between the probabilistic model and how robustly it responds to part variations (clearances) has been explored in this research.

The remainder of this paper is organized as follows; the problem description is introduced in section 2. Section 3 introduces the research methodology. The experimental setup is presented in section 4. The results are described in section 5 and a set of conclusions are drawn in section 6.

## 2. Problem Statement

The assembly process is generally split into two sub-tasks: gross motion and fine motion. In general, a gross motion is subject to no constraints in the environment, while during fine motion, the parts' movements are tightly constrained by the assembled parts' geometry. In this motion, a small error in a movement might cause an extensive force interaction leading to a failure of the assembly process. Hence, a force-based control is required to identify the CS and control the robot accordingly. In this context, the problem of CS recognition can be described as a classification problem, in which the F/T components are the raw data input **F** ∈ ℝ^*N*×6^ (three forces and three torques components in *x* − *y* and *z* directions) (Equation 1), where *N* is the number of samples, and the output is **Y** ∈ ℝ^*N*×1^, where **Y** is a pre-defined CS. Accordingly, the goal of the CS model is to identify the contact state of a PiH assembly process.

(1) $$\begin{array}{r} \begin{array}{c} \text{F}=\left[f_{0},\ldots ,f_{N}\right]\\ {\text{f}}_{i}=\left[F_{x},F_{y},F_{z},T_{x},T_{y},T_{z}\right]\\ i=0,\ldots ,N \end{array} \end{array}$$

Accordingly, the classification problem can be described as identifying a mapping function *h*(**F**, **Y**) that maps the given force measurements into a CS ($\text{F}\overset{h\left(\text{F},\text{Y}\right)}{\to }\text{Y}$).

## 3. Methodology

The methodology adopted in this research relies on dimensionality reduction and symbolic representation of multi-dimensional F/T signals, which aims to recognize the CSs of an assembly process. In order to capture the CSs of a PiH insertion, the force/torque time-series data is recorded, filtered, normalized, its dimensionality reduced and the resulting time-series is represented in a string of symbols. The mapping of these time-series data can be performed under the assumption that the normalized time-series is Gaussian (Lin et al., 2003). Each symbol in the resultant string is labeled to match a member from a pre-defined CS set. The resultant strings and their associated labels set are used to train an HMM to capture the assembly process sequence.

**Figure 1:**
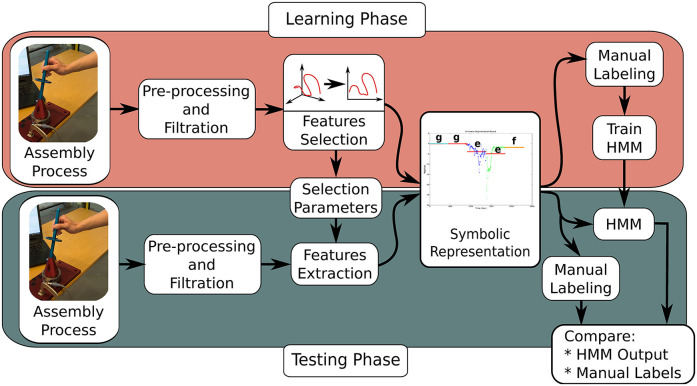
Overview of the proposed approach (training and testing phases).

The training approach adopted for this research is shown in Figure 1. The first step involves filtering and scaling F/T features using a low-pass filter and magnitude normalization, respectively. The data is projected into a new sub-space which maximizes the data variation and reduces dimensionality and noise using PCA. After that, the time-series is transformed to their symbolic representations. The symbolic representation is being assigned in two steps. Firstly, the time-series is segmented using Piecewise Aggregate Approximation (PAA) or K-means. Secondly, each segment from the previous step is being represented by a symbol based on its location in a normal distribution.

To verify the resulting models, unseen test sets were used. The accuracy of the trained models was measured based on a confusion matrix^1^. The pre-processing, feature transformation and symbolic representation stages of the research methodology are explained in more detail in the following sub-sections.

### 3.1. Pre-processing

The pre-processing consists of two stages, i.e., filtration and normalization, which are explained as described below.

- Filtration: The F/T signals are subject to electromagnetic noise which severely affects the F/T signal. It is noticeable that the raw data from the F/T sensor contains random fluctuations, burrs and spikes. Shielding of sensors and their wiring can partially solve this problem. However, this is not always practical. In Wu et al. (2014), a comparison amongst different filters to alleviate the noise effects on F/T signals is presented. A performance measure, called stability index, was used to evaluate those different filters. In conclusion, it was recommended to use an FIR filter together with a Double-Threshold Filter (DTF). Hence, in this work, a finite impulse response (FIR) low-pass filter with DTF was adopted for the data pre-processing step of the F/T signal. The F/T signal was sampled at 500 Hz and filtered using a low-pass filter with 35 Hz cutoff frequency and DTF.
- Normalization: In order to capture and compare features that occur at different force levels on different trials, the force information during different trials needs to be normalized. Normalization is a powerful feature scaling method especially when the extreme values (minimum and maximum) of given features are unknown (Han, 2005; Jamali et al., 2015). On the other hand, the test data must be normalized based on the normalization coefficient of the training data.

### 3.2. Feature Transformation

Transformation can be perceived as a search algorithm that attempts to find a new set of features to make the machine learning problem easier (Liu and Motoda, 1998). PCA is one of the most common feature transformation tools that rely on allocating the directions that maximize the variation in the features' space (Sophian et al., 2003). The PCA is a mathematical tool used to analyse data sets based on their variations. One main characteristic of PCA is a reduction in dimensionality which often results from this tool. This dimensionality reduction involves the selection of features with maximum variation based on the accumulative-variance and a user-defined threshold (Calinon and Billard, 2008). The PCA threshold defined the amount of data which can be returned from the PCA after feature extraction.

### 3.3. Symbolic Representation

For the symbolic representation, the Symbolic Aggregate Approximation tool (SAX) was modified and employed in this research due to its simplicity. The SAX tool is a symbolic representation tool of time-series data that assigns the representation of numeric values based on Euclidean distance and discretization process (Lin et al., 2007). It also allows us to represent different time-series (various lengths) with the same number of symbols (Keogh et al., 2005). This property is of great importance in time-series alignments. The symbolic representation is achieved in two steps: time-series segmentation and segments mapping into symbols.

#### 3.3.1. Time-Series Segmentations

Time-series segmentation can be achieved using PAA or K-means segmentation. In this paper, a brief comparison between the PAA segmentation and the well-known K-means time-series segmentation is presented.

#### 3.3.2. Piecewise Aggregate Approximation (PAA)

The PAA splits time-series data with length *N* into *M* segments. This is very useful, especially in encoding temporal data during human demonstrations, where each trial has its different temporal properties (e.g., duration of each state). The PAA approximates a single time-series *S*(*n*) into a vector of segments' averages; ($\bar{S}=\left({\bar{s}}_{1},\ldots ,{\bar{s}}_{M}\right)$) for any random length (*M* ≤ *N*), where each ${\bar{s}}_{i}$ is calculated as shown in Equation (2).

(2) $$\begin{array}{r} {\bar{s}}_{i}=\frac{M}{N}\overset{\frac{N.i}{M}}{\underset{t=\frac{N}{M}\left(i-1\right)+1}{\sum }}S\left(n\right) \end{array}$$

Accordingly, the resulting time-series $\bar{S}\left(n\right)$ is shown in Equation (3).

(3) $$\bar{S}(t)=\{\begin{array}{cc} {\bar{s}}_{1} & 0\leq t<\frac{N}{M}\\ \vdots & \vdots \\ {\bar{s}}_{i} & \frac{N}{M}(i-1)+1\leq n<\frac{N}{M}i\\ \vdots & \vdots \\ {\bar{s}}_{M} & \frac{N}{M}(M-1)+1\leq n<N \end{array}$$

The PAA represents a single time-series (1D) data into a sequence of averages $\bar{S}$. However, applying the PAA on multi-dimensional time-series will result in a sequence of vectors ($\left(\bar{S}\right)$) where each element in the vector is a *D* dimension (selected features) corresponding to each time-series from the PCA. In this research, it is required to represent the multi-variable time-series with a single sequence of symbols. Hence, the PAA needed to be modified for the multi-variable time-series to be represented using a one-dimensional sequence of averages. Accordingly, a further dimensionality reduction is needed on the PCA result. This reduction can be performed using the average of the multi-dimensional data over different sectors of the PAA. Another alternative is to employ the norm of the multi-dimension data. In this paper, the norm method was used since it can be physically interpreted as the magnitude of the feature vector. Equation (4) represents the modified PAA using norm, where $\bar{S}\left(n\right)$ is a vector of data at time *n*.

(4) $$\begin{array}{r} {\bar{s}}_{i}=\frac{M}{N}\overset{\frac{N.i}{M}}{\underset{n=\frac{N}{M}\left(i-1\right)+1}{\sum }}||\text{S}\left(n\right)|{|}_{2}^{2} \end{array}$$

The result from the PCA and its corresponding PAA results are shown in **Figure 11**. Then, each segment is mapped into a symbol as illustrated in the next section.

#### 3.3.3. K-means Time-Series Segmentation

One of the simplest and most popular methods to overcome the clustering problem is the K-means algorithm (De la Torre and Kanade, 2006). K-means clustering splits a set of *N* samples (e.g., time-series) into *M* groups by maximizing the ratio amongst different clusters and the variation of each cluster. A K-segmentation of a time-series *S* is a sequence of mean values $\bar{S}$. Under consideration of the given context, the K-means problem can be described as the problem of allocating a segments boundaries (temporal information) (Vlachos et al., 2003). Equation (5) depicts the interval definition over all segments. The input for the K-means algorithm is the norm value of the multi-dimensional data from the PCA and the temporal information. The output is a time-series $\bar{S}\left(n\right)$, where each data point is represented by the centroid of the *i*th cluster/segment. The drawback of using K-means is its dependence on the initial estimation of the centroid and the number of clusters, which means that K-means might have different segmentation results for different initialization.

(5) $$\begin{array}{r} \begin{array}{c} {\bar{s}}_{i}\in \left\{s\left(t_{a}\right),\ldots ,s\left(t_{b}\right)\right\}\\ {\left(t_{a}\right)}_{i}=\underset{t}{min}\;{\bar{s}}_{i}\\ {\left(t_{b}\right)}_{i}=\underset{t}{max}\;{\bar{s}}_{i} \end{array} \end{array}$$

Where ${\bar{s}}_{i}$ is the *ith* segment that starts at (_*t*_*a*_)*i*_ and ends at (_*t*_*b*_)*i*_. The accuracy of the K-means was tested under a different number of clusters (as explained in section 5), and the number of segments with the best accuracy was selected. Based on PAA and K-means, the different time-series (trials) with different length *N* were represented using the same number of segments. The resulting segments have a unity magnitude. After that, each segment is represented by a single symbol based on its location in the normal distribution. It is worth mentioning that the number of K-means centroids and segments in the PAA were determined based on the elbow method, where classifier accuracy was tested with a different number of centroids and segments.

#### 3.3.4. Segmentation Mapping

Having transferred the time-series data into segments (PAA or K-means), a further transformation must be applied to achieve the symbolic representation. Under the assumption that the normalized time-series is Gaussian as highlighted in section 3, the mapping of segments into symbols adopted in this paper has been introduced in Lin et al. (2007). In which, the outputs from the PAA and K-means are mapped into a series of symbols using predetermined “breakpoints” that produce equal-sized areas under a Gaussian curve with (N(0,1)). The maximum number of breakpoints supported by the tool developed in Lin et al. (2007) is 12, these were adopted in this research to reduce the effect of the discretization error.

**Figure 2:**
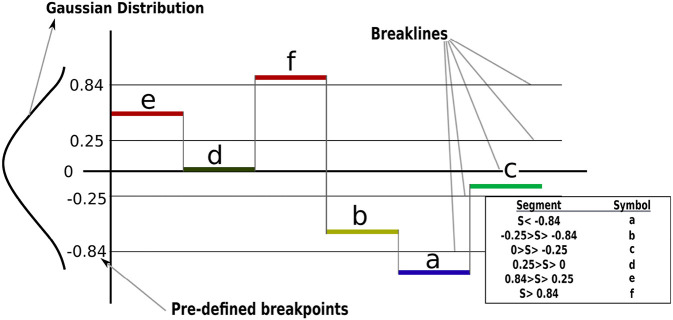
Segments mapping into a symbolic representation of time-series data. For example, the time-series is segmented into six segments and each segment mapped into a symbol based on its location with respect to the Gaussian distribution (breaklines).

Figure 2 shows how a segmented signal based on subsection 3.3.1 mapped into symbols based on their location with respect to the predetermined breakpoints. Then, the force-time-series for the different trials are represented in a single sequence of symbols; e.g., (*Symbols* : = {*jjjiihcbaafff*}), where a sequence of symbols encodes the CSs (hidden). From Figure 2, any segment that appears lower than the break line at −0.84 will have the symbol *a* throughout the trials, the force/torque time-series were represented using the same number of segments, even though the insertion process durations were different for each trial. Similar stages were represented using similar symbols using a normal distribution. For example, *J* and *I* are representing no-contact stage and *H*, and *C* are representing Chamfer-Crossing stage. Accordingly, different trials can be aligned using their corresponding symbols. The goal is to capture the relation between the recognized pattern (symbols) and the CSs. One possible solution for such a problem is to use an HMM.

#### 3.3.5. Manual Labeling of PiH Insertion

**Figure 3:**
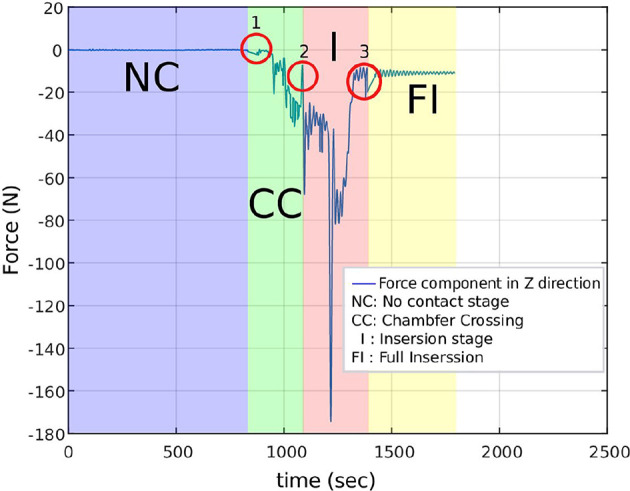
Manual labeling of the PiH insertion process based on *F*_*z*_ force component.

**Figure 4:**
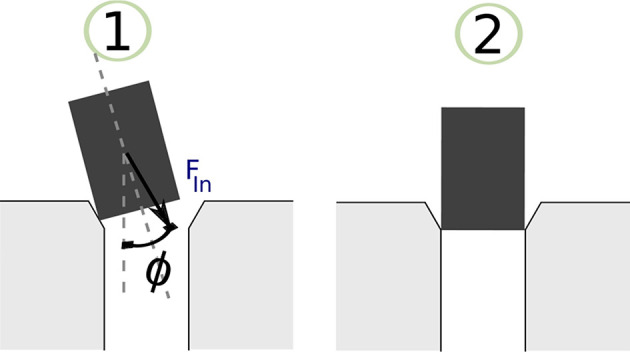
Chamfer-Crossing stage.

**Figure 5:**
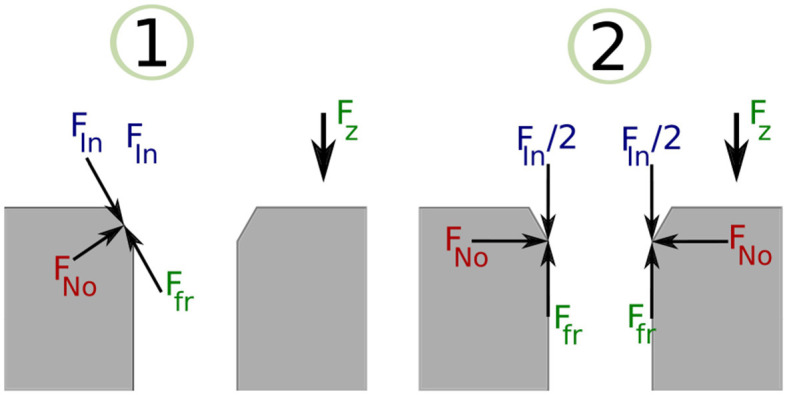
Chamfer-Crossing PiH stage: force analysis.

The resulting sequence of symbols introduced in the previous section is vague, and an expert should manually label it. A manual labeling process was performed based on analysing the *F*_*z*_ component of the data sets, because the F/T sensor is stationary and most of the force variation occurs on *z* direction. Figure 3 illustrates the *F*_*z*_ component and the corresponding process stage based upon specific features of the *F*_*z*_ trend. The red circles indicate the start of a new stage and the end of the previous stage. The first circle highlights the force trend as the first contact occurs and the Chamfer-Crossing starts, as shown in stage 1 of Figure 4. After this, the operator starts correcting the angular error (the angle between the hole axis and insertion force direction). Once the angular error approaches zero (approximately), the friction force reaches its maximum due to further contact, which causes an overshoot in the force trend. This overshoot is highlighted in the second circle in Figure 3. Stage 1 of Figure 5 shows the force analysis when the first contact point occurs and Equation (6) explores the force analysis at this stage. Stage 2 of Figure 4 outlines the initial alignment, where the friction force *F*_*fr*_ is doubled whilst the insertion force *F*_*In*_ stays relatively constant as shown in Equation (7). This alignment explains the spike at the end of the Chamfer-Crossing (Figure 3, second circle). The insertion process then commences, and the peg is pushed fully into the hole. Once the peg is fully inserted in the hole, the operators release the peg causing a relaxation in the insertion force. This results in the small spike in the third circle in Figure 3 which indicates the end of the insertion process. It is worth mentioning that these characterizations were observed in all PiH insertion trials. Therefore, the CS set (**Y**) is defined based on the PiH assembly stages as follow: (**Y** = {*NC, CC, I, FI*}); where *NC* is No Contact State, *CC* is Chamfer-Crossing, *I* is Insertion, and *FI* is Full Insertion.

(6) $$\begin{array}{r} F_{z}=\left(F_{fr}+F_{No}-F_{In}\right)\text{ cos}\left(\phi \right) \end{array}$$

(7) $$\begin{array}{r} F_{z}=2\;F_{fr}-F_{In} \end{array}$$

The manual labeling of the symbolic representation was applied to enhance the process of obtaining human skills and to highlight the physical meaning of the discovered patterns. Also, the labeled data is only used for training and testing purposes and is not required for later interpretation of new PiH processes once the model has been verified.

#### 3.3.6. Hidden Markov Model (HMM)

Once the F/T information is transformed into strings of symbols which represent the temporal information of the PiH assembly process, an HMM was used to encode the temporal information and detect the pattern of each CS. Accordingly, each assembly trial (human demonstration) was represented in a string of symbols. The resulting strings were manually labeled by combining each symbol in the strings with one element from the CS set as explained in subsection 3.3.5. This resulting dataset can be represented as shown in Equation (8), which is used to train the HMM models. The same data set were used to initialize emission and transition matrix of the HMM using the Baum-Welch (BM) algorithm (Hochberg et al., 1991).

(8) $$\begin{array}{r} X_{Training\;Data}=\ldots \left(d,CC\right)\left(e,CC\right)\left(b,I\right)\left(c,I\right)\left(a,I\right)\ldots \end{array}$$

**Figure 6:**
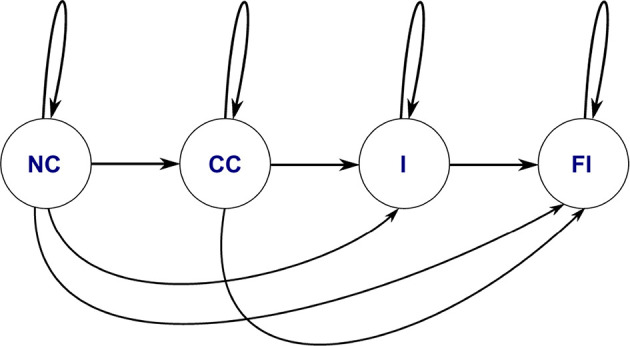
HMM topology of the PiH insertion process based on the given dataset in Equation (8).

Figure 6 depicts the typology of the HMM used for the symbolic representation of each trial. This HMM encoded the PiH assembly skills, which was represented in a sequence of symbols. The HMM was trained using the string of symbols (as the observation) and CSs (as hidden states) to predict the new cases.

To summarize, the proposed approach is composed of three main stages. The first stage is segmentation which discovers the spatial structure within the data. Secondly, the symbolic representation reduces the high dimensional time-series data into one-dimensional data. The third stage captures the temporal knowledge embedded in the symbolic representation. For testing purposes, the labels for the randomly chosen test data sets were generated based on the trained model without using manual labeling. The results were then compared with the manual labels to evaluate the accuracy of the trained model.

## 4. Experimentation Setup and Data Acquisition

**Figure 7:**
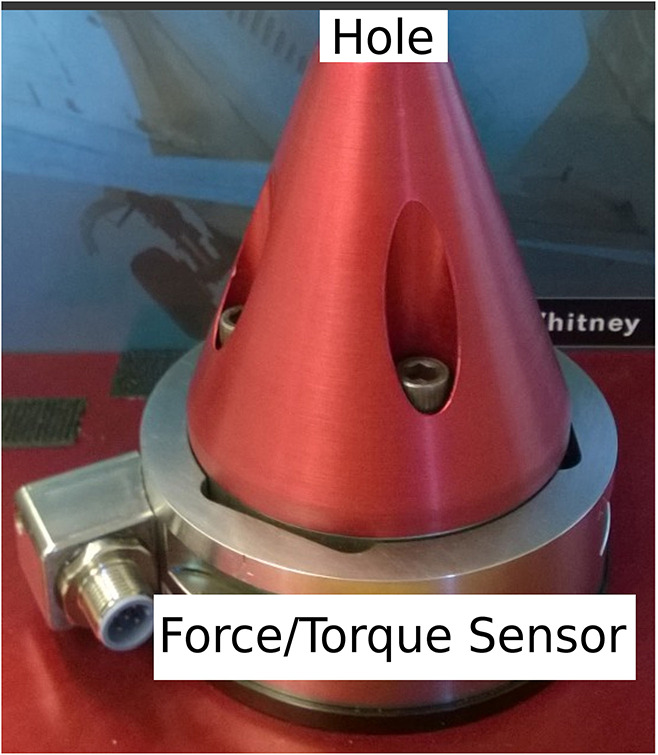
Hole and F/T sensor.

**Figure 8:**
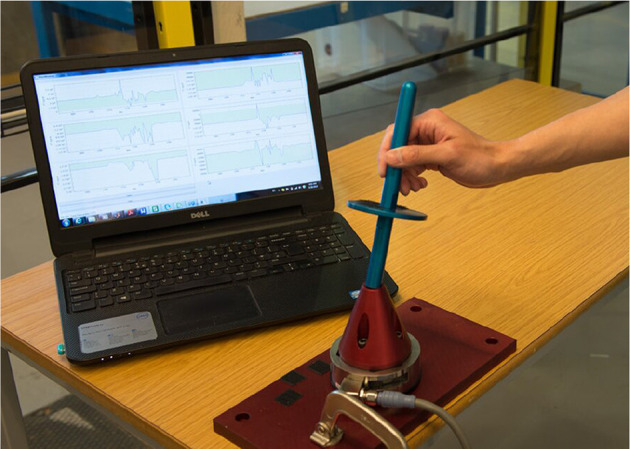
The experiment setup during PiH insertion.

The experimental setup shown in Figure 7 was used to collect data from different human operators performing a PiH assembly process. This setup was composed of a six-axis F/T sensor, a hole with a diameter *D* of 16.20 *mm*, and two round mating parts with different diameters (*Peg 1* and *Peg 2*). Where, the diameter of *Peg 1* is 15.98 *mm* and the diameter of *Peg 2* is 15.87 *mm*. Figure 8 depicts one trial of the insertion process. The F/T data has been recorded while the human operators performed the assembly task.

A total number of 60 experiments were carried out with three different operators. Each operator performed 20 trials, to capture a wide range of human skills and variation in the initial position of the peg. Each trial contains on average 1,500 data points of F/T signals. The collected data were randomly split into training data (80% ≈ 48 trials) and test data (20% ≈ 12 trials). The six-dimensional time-series data (features) recorded by the F/T sensor was reduced to two-dimensional data using PCA. Then, the two-dimensional time-series data were reduced to 1*D* data by taking their norm value in the modified form of the PAA or K-means. After that, the segmented data were represented by a string of symbols. Those strings were labeled and used to train an HMM to discover the temporal aspects of the assembly process.

The quality of the classifier based on the HMM was evaluated using an unseen test set. This process was repeated four times to get an average performance of the classifier based on the proposed approach (see section 3). Figure 1 depicts the evaluation process using the test set. It is worth mentioning here that the same mixing matrix ζ and the normalization coefficients of the training stage were used to pre-process test data; under the assumption that statistical properties of the test data are unknown, during the evaluation process. Then, the accuracy of the HMM model calculated with respect to the label data.

## 5. Results and Discussion

**Figure 9:**
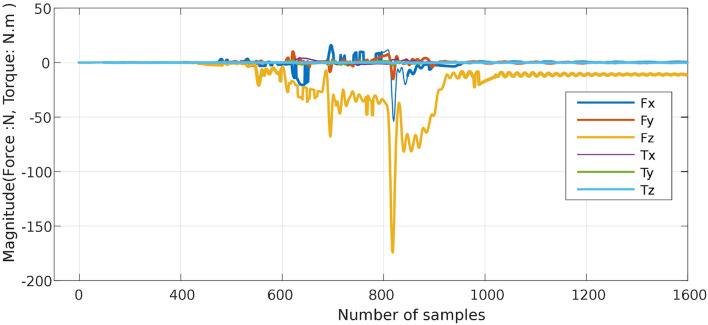
Six-dimensional F/T signal during PiH assembly (original input data ℝ^6^).

**Figure 10:**
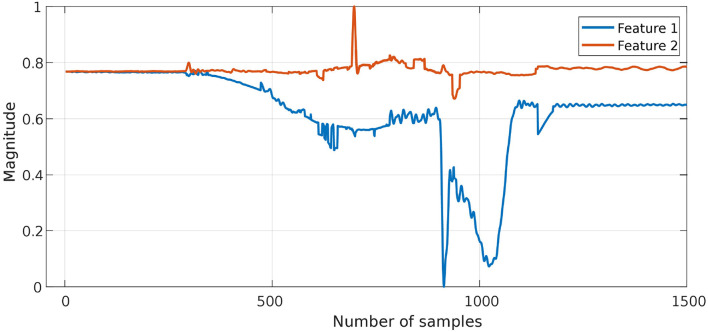
The transformed F/T data (ℝ^2^) after features transformation in the latent space using PCA. The accumulative-variance threshold was 90% of the total variance of all signals.

**Figure 11:**
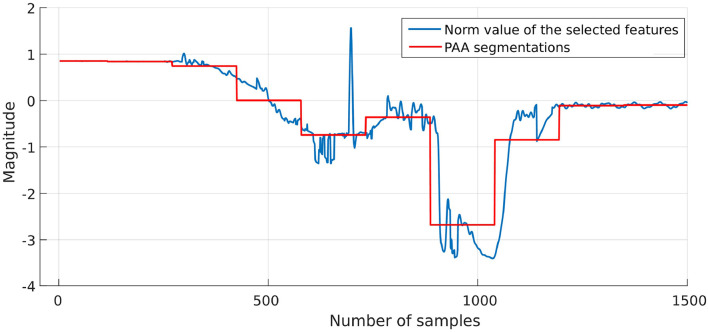
PCA and corresponding PAA result.

**Figure 12:**
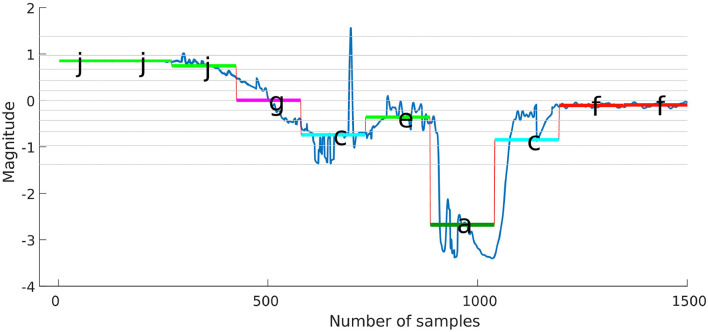
Symbolic representation with five segments using PAA.

**Figure 13:**
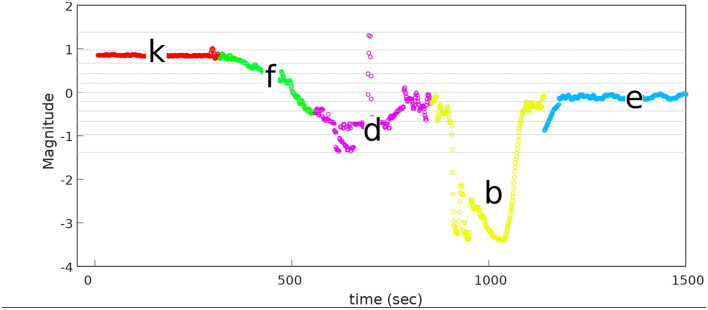
Symbolic representation with five segments using K-means.

The proposed approach was designed to recognize CSs during the assembly task efficiently, and then it was evaluated using PiH insertion problem as discussed before. Next, fitted models were evaluated as described in section 4. At the beginning, the collected during PiH insertion were six-dimensional (**X** ∈ ℝ^6^) as shown in Figure 9, while the transformed data is two-dimensional (${\text{X}}_{red}\in {\mathbb{R}}^{2}$) as illustrated in Figure 10, which indicates the PCA selected features from the raw data in Figure 9. The resulting PCA components were signals that have an accumulative variance that is higher than 90% of the total variance. The selected features were segmented using the modified PAA and K-means. The modified PAA and symbolic representations of the time-series data are shown in Figure 11. Figure 12 depicts the symbolic representation results based on the PAA segmentations. Figure 13 illustrates the K-means segmentations and the corresponding symbolic representation, where each color represents a segment.

In order to compare the segmentation approaches (PAA and K-means) and to determine the suitable number of segments for each segmentation approach, the symbolic representation was carried out based on PAA and K-means separately with a different number of segments. A critical difference between the PAA and the K-means segmentation is that the temporal and spatial features are crucial for the K-means segmentation. In contrast, PAA splits data into segments of equal length (temporal length) without taking spatial data into account. After that, temporal knowledge can be captured using HMM.

**Figure 14:**
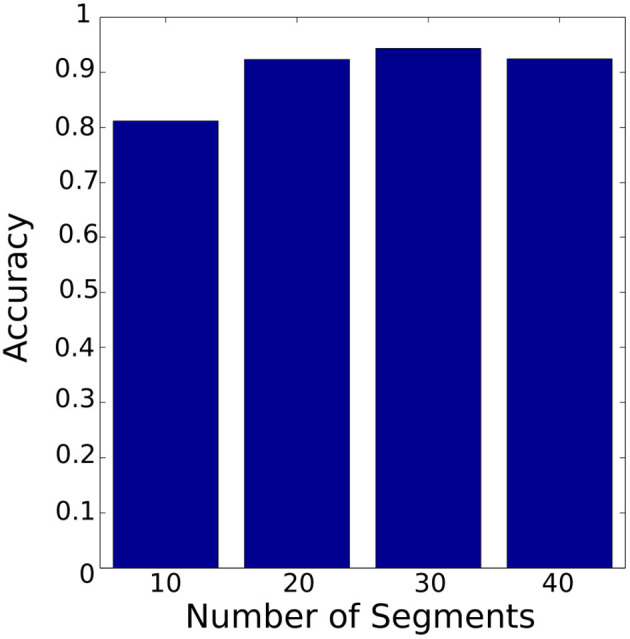
Classifier accuracy with PAA segmentation using different number of segments. The best accuracy was achieved with 30 segments.

**Figure 15:**
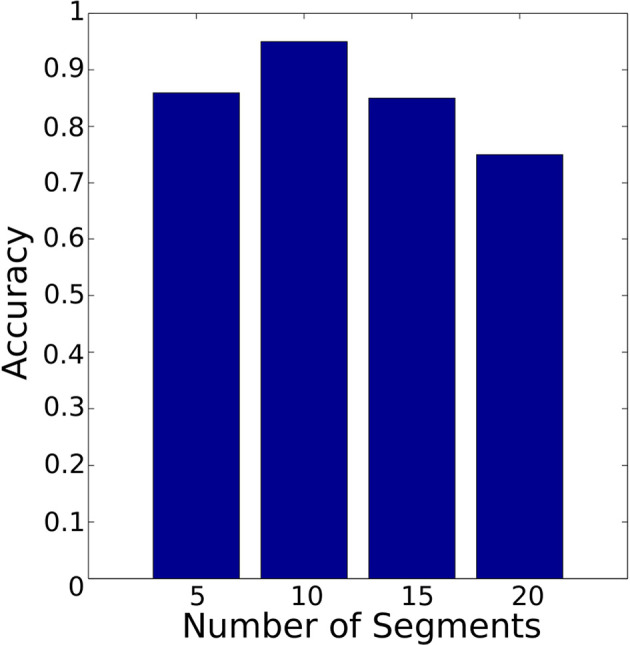
Classifier accuracy with K-means segmentation using different number of segments. The best accuracy was achieved with 10 segments.

Figure 14 shows the accuracy of the HMM model based on PAA segmentation. The highest accuracy is 94% using 30 segments with 0.88 s computational time. In comparison Figure 15 illustrates the accuracy of the HMM model based on K-means segmentation. The highest accuracy is 95% using 10 segments with 11.86 s. Those results indicate that models generated based on K-means segmentation do not require a large number of segments to achieve high accuracy. The models created using PAA require a large number of segments to improve the accuracy of the model. The model based on K-means segmentation achieved higher accuracy with a lower number of segments. This requires an extensive search until it converges to the optimal segmentation with resulting segmentation depending on the initial estimation of the segments' centroids. Surprisingly, the accuracy decreased dramatically with an increased number of segments. This shows there is no linear relationship between the number of segments and the accuracy. Therefore, an optimal number of segments needs to be identified requiring an additional iterative process. Conversely, the models generated using the PAA are more robust and do not request an iterative search. Also, the PAA segmentation returns the same segments for the same trial repeatedly. The results presented so far correspond to the data collected during the insertion of *Peg 1* without considering the variation in clearance.

Another important aspect in the PiH assembly process is the clearance, where assembly of tight clearance parts is more difficult than loose clearance parts. In order to test the models for different clearances, two models; *model 1* and *model 2*, were trained separately using the sequences captured during the assembly of *Peg 1* (tight) and *Peg 2* (loose), respectively (see section 4). Both the models were tested to explore the relationship between the accuracy of CS recognition and the clearances.

To evaluate the classification accuracy of the two models both models were tested with unseen labeled data (for assembling *Peg 1* and *Peg 2*). The resulting accuracy is shown in the confusion matrices in Tables 1, 2. Table 1 shows the confusion matrix of the HMM trained using the PAA with 30 segments (*model 1*). It can be observed from the table that the *CC* stage is being the least accurately classified.

**Table 1:** Confusion matrix of *model 1* for *Peg 1* clearance *c* = 0.11 *mm*, where NC, no contact; CC, Chamfer-Crossing; I, insertion; FI, full insertion.

	**NC**	**CC**	**I**	**FI**
NC	83	7	0	0
CC	0	14	1	0
I	0	0	50	0
FI	0	0	0	78

**Table 2:** Confusion matrix of *model 2* for *Peg 2* with clearance *c* = 0.17 *mm*.

	**NC**	**CC**	**I**	**FI**
NC	82	5	0	12
CC	0	10	0	36
I	0	21	31	0
FI	0	0	12	31

Table 2 shows the confusion matrix of the HMM trained using the PAA with 30 segments (*model 2*). An analysis of the result reveals that the misclassification of the *CC* stage that happens due to the static friction that occurs directly after the first contact. Also, the force level during this stage overlaps with the force level at the full-insertion stage which means that the mapping process will assign the same symbols for both stages (*CC* and *FI*).

The overall accuracy of *model 1* and *model 2* are 94 and 64%, respectively. Therefore, the trained models derived from the insertion of the larger clearance peg have a lower accuracy than the model based on the tighter clearance peg. The reason behind this is that the tighter clearance creates a stronger boundary amongst the CSs. Nevertheless, parts with larger clearances can partially change their contact state without causing distinguishable variation in the F/T signal which makes the recognition of distinct CS more difficult.

Additionally, the model with higher accuracy *(model 1)* was used to recognize the assembly CSs of *Peg 2* to examine the robustness against clearance variation. The performance of CS recognition based on *model 1* is illustrated in the confusion matrix as shown in Table 3. The overall accuracy reduced from 96 to 82.4%. Though, the accuracy of *model 1* on *Peg 2* is still better than the accuracy of *model 2* on *Peg 2*, this shows that *model 1* is quite robust against clearance variation.

**Table 3:** Confusion matrix of *model 1* validated using observation of *Peg 2*.

	**NC**	**CC**	**I**	**FI**
NC	82	12	0	0
CC	0	9	1	0
**I**	0	21	31	0
**FI**	0	0	11	71

The results generated were compared with the most relevant work from the literature. In this regard, the method introduced by Jamali et al. (2014) achieved an overall accuracy of 81%, and 85% for rotation about the x-axis and the y-axis, respectively. The HMM-PAA models proposed in this paper has an accuracy of 94% and is, therefore, an improvement. However, to ensure that the accuracy is not due to chance, the datasets from all users for *Peg 1* and *Peg 2* have been combined and then randomly split 100 times into train and test data. The confusion matrices of the 100 times split using HMM-PAA and HMM-K-means are shown in Tables 4, 5, respectively. The average accuracy of the HMM-PAA model is (90 ± 1.38)%, while it was only (76 ± 1.45)% for the HMM-K-means model. Table 6 illustrates the overall accuracy, precision, and F-score of both HMM-PAA and HMM-K-means models. These numbers show better accuracy and robustness (precision) of the HMM-PAA in comparison with the HMM-K-means. The overall accuracy of the HMM-PAA was 90% with σ equals to 8.4%, while HMM-K-means has an accuracy of 76% with σ equals to 8.2%. This shows that the accuracy of both approaches has similar standard variation with different overall accuracy.

**Table 4:** Confusion matrix of HMM trained with PAA 30 segments.

	**NC**	**CC**	**I**	**FI**
NC	1,115	90	0	0
CC	65	180	25	0
I	0	20	500	130
FI	0	0	15	1,160

**Table 5:** Confusion matrix of HMM trained with K-means 10 segments.

	**NC**	**CC**	**I**	**FI**
NC	290	30	5	0
CC	90	60	10	0
I	0	55	135	65
FI	0	0	10	345

**Table 6:** Overall accuracy (100 times split) of the HMM models with PAA and K-means.

**Method**		**Accuracy (%)**	**Precision (%)**	**F-score (%)**
HMM-PAA	μ	90	85	84
	σ	8.4	7.5	7.5
HMM-K-means	μ	76	73	72
	σ	8.2	7.5	7.3

The proposed approach greatly reduces the required computation time, although it relies on multi-stage processes. Table 7 shows the computational complexity of the proposed approach in comparison with three similar research approaches, namely Jamali et al. (2014), Jasim et al. (2017), and Hannaford and Lee (1991), where *N*_*symbols*_ is the number of symbols, *K* is the number of original dimensions before the PCA, *M* is the number of segments, *N*_*sample*_ is the number of samples within the time-series and *D* is the number of selected features (selected dimensions based on the PCA). For the proposed approach with PAA, the worst case scenario occurs when the *N*_*symbols*_ is 12, and *M* is 30. In this case, the complexity of the HMM is the bottleneck; hence, the total complexity is $O\left(2KN_{samples}D\right)$. On the other hand, the worst case for the proposed approach with K-means occurs when the *N*_*symbols*_ is 12, and *M* is 10; however, the time complexity of the K-means is quadratic of the *N*_*samples*_, which was on average 1500 samples. Henceforth, the K-means is the bottleneck for this case, which explains the long execution time to recognize the CS in comparison with PAA. In comparison with the method introduced in Jamali et al. (2014), the complexity of MML-GMM, that was used to cluster the Force/Torque data), was $O\left(MN_{samples}D\right)$. Nevertheless, the complexity of the proposed approach was O(M), that only depends on *M*, while the complexity of MML-GMM depends on *M* multiplied by *N*_*samples*_ and *D*. The overall performance appears similar in both methods. However, the method proposed in Jamali et al. (2014) requires additional exploration stage (set of random movements on *x* and *y* direction) before starting the recognition stage. Henceforth, it might require a longer time until it converges. In Jasim et al. (2017) the EM-GMM were utilized without dimensionality reduction, which means that the complexity is $O\left(MN_{samples}D\right)$. While in the proposed approach the dimensionality reduction greatly reduced the number of features and the samples. Also, as shown in Table 7 the total complexity of the proposed approach is $O\left(2KN_{samples}D\right)$ which is less than the complexity of the EM-GMM utilized in Jasim et al. (2017) as long as 2*K* < *M*. Finally, the computational complexity of the HMM presented by Hannaford and Lee (1990) was $O\left(N_{samples}^{2}D\right)$, which is higher than the total complexity of the proposed approach.

**Table 7:** Computational complexity comparison.

	**Proposed approach (PAA)**	**Proposed approach (K-means)**	**MML-GMM (Jamali et al., 2014)**	**EM-GMM (Jasim et al., 2017)**	**HMM (Hannaford and Lee, 1990)**
PCA (after training)	$O\left(2KN_{samples}D\right)$	$O\left(2KN_{samples}D\right)$	$O\left(2KN_{samples}D\right)$	–	–
GMM	–	–	$O\left(MN_{samples}K\right)$	$O\left(MN_{samples}D\right)$	–
PAA	$O\left(N_{samples}\right)$	–	–	–	–
K-means	–	$O\left(N_{samples}^{2}\right)$	–	–	–
HMM	$O\left(MN_{symbols}^{2}\right)$	$O\left(MN_{symbols}^{2}\right)$	$O\left(MN_{symbols}^{2}\right)$	–	$O\left(DN_{samples}^{2}\right)$
Total	$O\left(2KN_{samples}D\right)$	$O\left(N_{samples}^{2}\right)$	$O\left(2KN_{samples}D\right)$	$O\left(MN_{samples}D\right)$	$O\left(DN_{samples}^{2}\right)$

## 6. Conclusions

This paper proposed a method to capture human skills during the PiH assembly process utilizing a learning algorithm to encode the assembly process. The proposed algorithm was based on a symbolic representation of F/T signals in the presence of geometrical variation of the assembled parts. This approach is capable of recognizing the CSs of PiH assembly process based on a symbolic representation of force and torque information. It can accommodate variations in the insertion force levels and compensate for process noise. The main benefits of this method are its simplicity and minimal pre-knowledge requirements about the geometrical information of the mating parts.

During the symbolic representation, two segmentation approaches, i.e., the K-means and the PAA, were investigated for their effectiveness. It was found that a higher accuracy of CS recognition can be achieved with a small number of segments when using K-means to segment the F/T time-series whereas the models trained based on the PAA segmentation require a higher number of segments. The model which was trained based on the K-means resulted in an accuracy of 70% with 10 segments with an 12 s computational time. The model generated based on the PAA resulted in an accuracy of 90% accuracy with 30 segments with 0.95 s computational time. The K-means requires more computational effort due to its iterative nature, whereas the PAA is a simpler and faster segmentation procedure. The use of the PAA in the symbolic representation reduces the required computational effort and increases the robustness of the model against process noise.

In this research, the robustness of the trained models was examined by varying part mating clearances. The results showed that the CS recognition is more accurate for tight clearance mating. This observation implies that there is an inverse relationship between the clearance and the accuracy of the CS recognition. This is due to the higher physical constraints in a tight clearance insertion process, providing a better-defined boundary that separates the consecutive CSs. The model trained based on tight clearances peg is more robust against geometrical variation.

The availability of robust and computational efficient representations is an essential precursor for imitation learning. The proposed approach achieves those two goals. However, it heavily relies on approximation and dimensionality reduction that might remove essential features from the force trend. Accordingly, the proposed approach might be not suitable for applications that require high accuracy, such as textile recognitions. Future work will consider the transformation of the trained models to an industrial robot by extending the proposed approach to a complete imitation learning framework. It is believed that humans often rely on visual perception to perform handling task. Hence, the proposed methods can be extended to include visual features that might improve the models' accuracy.

## Data Availability Statement

All datasets generated for this study are included in the manuscript/supplementary files.

## Author Contributions

AA-Y and YZ conducted the design, the adopted methodology, and the experiment. All authors conducted the data collection, data analysis, and results interpretation.

### Conflict of Interest

YZ was employed by Beijing Ewaybot Technology LLC. The remaining authors declare that the research was conducted in the absence of any commercial or financial relationships that could be construed as a potential conflict of interest.

## Footnotes

^1^Performance of classification is commonly evaluated using the data in confusion matrix.
